# The psychological impact of genocide on the Yazidis

**DOI:** 10.3389/fpsyg.2023.1074283

**Published:** 2023-03-29

**Authors:** Jan Ilhan Kizilhan, Thomas Berger, Laura Sennhauser, Thomas Wenzel

**Affiliations:** ^1^University of Duhok, Duhok, Iraq; ^2^Institut for Transcultural Health Science, Cooperative State University Villingen-Schwenningen, Stuttgart, Germany; ^3^Department of Psychology, Clinical Psychology and Psychotherapy, University of Bern, Bern, Switzerland; ^4^World Psychiatric Association, Scientific Section on Psychological Aspects of Persecution and Torture, Geneva, Switzerland

**Keywords:** refugee camps, psychological stress, genocide, trauma, Yazidi

## Abstract

**Background:**

The genocide against the Yazidis by the Islamic State of Iraq and Al-Sham (ISIS) in the Sinjar area of Northern Iraq has costed many lives and has also caused a psychological long-term impact in this minority. This impact can be seen among individual survivors. Additionally, there is a large number of direct and indirect victims and for this reason, the impact can also be observed on the level of the group and society in this region at large.

**Methodology:**

The research examines three different population groups (Yazidis members who had been exposed to violence by terrorist group actions, those not exposed to this experience as they were living in an area not directly exposed to ISIS violence, and a control group of non – Yazidi general population members). In total, 425 participants (age range 15–78) took part in the study and participated in interviews using standard scales to measure general physical and mental health.

**Results:**

The results demonstrate that psychological stress and suicidality are higher among the Yazidis survivors of violence than in the other Yazidi participants.

**Conclusion:**

Psychological disorders after a genocide and war in post-conflict populations should receive more attention in the planning of mental health care and prevention and should be seen as a major problem, especially in camp settings and displaced persons besides the usual increased prevalence of posttraumatic stress and other disorders covered by research so far in this context.

## Introduction

The Yazidis are a small ethnic-religious minority group which is spread over several Middle Eastern countries including Iraq, Syria, Armenia, Georgia and Turkey (Maisel, 2008). They speak Kurdish and most of them consider themselves ethnic Kurds, although some communities prefer identifying themselves as Yazidis with their own ethnic and religious identity (Kreyenbroek and Omarkhali, 2016). There are about 800,000–1,000,000 Yazidis worldwide (Cetorelli et al., 2017), the largest group of about 400,000 Yazidis lives in Northern Iraq in the area of Mount Sinjar in Nineveh governorate (Maisel, 2008). They practice one of the oldest religions, notably Yazidism, which contains elements of Islam, Christianity, Judaism and Zoroastrianism (Cetorelli et al., 2017) and is passed on orally (Kizilhan, 2014). Many derogative prejudices exist against the Yazidi community, one of them being the wrong accusation of engaging in “devil-worshipping” (Maisel, 2008). For this reason, they are considered as “Devil Worshippers” and not regarded as “Followers of the Book” by radical Muslims (Kizilhan, 2017a). Yazidis have been victims of persecution, forcible conversion to Islam and systematic murder for many centuries. In the last 800 years, 74 genocides against this minority group have been perpetrated (Kizilhan, 2017a). The Yazidis have been particularly affected by massacres due to their religion and their secluded settlement areas in the Sinjar Mountains (Kizilhan et al., 2020a) and the lack of protection (Maisel, 2008). For these reasons, it can be argued that the group as a whole has been exposed to a “genocidal environment” with far reaching, also trans-generational impact (Kizilhan et al., 2022), while other groups in the region were not exposed to transgenerational, multiple genocides.

In August 2014, ISIS attacked the Sinjar region of Northern Iraq (Independent International Commission of Inquiry on the Syrian Arab Republic, 2016). The Yazidis were forced to seek refuge on Mount Sinjar (Independent International Commission of Inquiry on the Syrian Arab Republic, 2016). More than 3,000 of them were killed and almost 7,000 kidnapped (Cetorelli et al., 2017). Thousands of Yazidis were tortured, suffered inhuman and degrading treatment, were held hostage, or were forced to convert to Islam. Young boys were separated from their families and placed with ISIS fighters and women and young girls were raped and sold on Arab markets (Cetorelli et al., 2017; Kizilhan, 2017a). Many of those who managed to flee remained trapped in tremendous circumstances without water, food, shelter, and enduring extreme temperatures and were killed or kidnapped if they could not flee in time. They were evacuated between the 9^th^ and 13^th^ of August and finally managed to flee through Syria into the KRI (Yazda, 2017). The majority was displaced in camps in the KRI, others settled in camps in Syria or Turkey (Daloglu, 2014; Sidky and Rummery, 2014). While some Yazidis returned to their home villages (Spencer, 2015), many of them found asylum in foreign countries including Germany or Turkey (Tekin et al., 2016; Kizilhan, 2017a). But the biggest part still lives as internally displaced people (IDP) in so-called IDP camps or informal settlements (Yazda, 2017). Most of the severely traumatized Yazidi women live in one of the 24 refugee camps near Dohuk and Zakho in the KRI. Each camp consists of up to 28,000 IDP (Kizilhan and Noll-Hussong, 2017). There are an estimated 360,000 Yazidis who have lived in camps for IDP in the KRI (Yazda, 2017). Not only did the Yazidis experience their own individual trauma, but they also experiences a collective and transgenerational trauma which is passed on to the next generations (Kizilhan and Wenzel, 2020).

The situation in these IDP camps in Iraq and also, in general, the situation in refugee camps is often extremely precarious (Cetorelli et al., 2017). A systematic review of psychiatric disorders among forcibly displaced people in conflict settings demonstrated prevalence rates mental disorders much higher even than in more general post-conflict and war-zone populations, for PTSD up to almost 90% as well as for depression and anxiety disorders of up to 80% (Morina et al., 2018). Surveys show that remoteness, poor connection and poverty in the camps lead to a lack of supply and care (Yazda, 2017; Jäger et al., 2019).

Recently, a new set of problems arose due to the current COVID-19 pandemic which has exacerbated the prior psychological stress. Social distancing is almost impossible in the overcrowded IDP camps and hygiene practices are difficult to apply (Raju and Ayeb-Karlsson, 2020). A study by Kizilhan and Noll-Hussong (2020) compared the prevalence rates of mental disorders before COVID-19 and shortly after COVID-19 outbreak of Yazidis living in IDP camps in the KRI. It was found that PTSD rates increased significantly to up to 58% among women and up to 47% among men. Also depression, anxiety, dissociation, somatoform disorders and suicidal ideas increased (Kizilhan and Noll-Hussong, 2020).

Prevalence rates of mental disorders are estimated to be even higher in survivors of rape, military action, captivity, internment for ethnic or political reasons, or genocide (American Psychiatric Association, 2013). For example, research conducted in Rwanda (Schaal and Elbert, 2006) and Bosnia (Weine et al., 1995) shows that genocidal atrocities have severe long-term effects on the people concerned. Forty-four percent of Rwandan adolescents suffered from PTSD 10 years after genocide, but the percentage of the adolescents presenting some of the PTSD-related symptoms is even higher (Schaal and Elbert, 2006). Among the Bosnian refugees, up to 65% suffered from PTSD and 35% from depressive disorders a few months after their resettlement in the United States (Weine et al., 1995).

During genocide, sexual violence against women is a serious problem and rape is used frequently as war tactic because of its devastating physical and psychosocial consequences on the individuals and also on the whole community (Ruby Reid-Cunningham, 2008). When rape is committed on a mass scale, consequences are even more serious (Ruby Reid-Cunningham, 2008). This reality was illustrated by different studies conducted in Bosnia-Herzegovina, Rwanda, Sudan, Uganda and Congo where rape and extreme sexual violence were carried out against women (Amnesty International, 2004; Lončar et al., 2006; Cohen et al., 2009; Amone-P’Olak et al., 2016; Albutt et al., 2017).

Whether people develop a mental disorder after a potential traumatic event (PTE) depends on various risk factors (Kizilhan et al., 2020a). First and foremost, higher exposure to traumatic experiences (Steel et al., 2009) and post-migration stress (Bogic et al., 2015) seem to play a particularly important role. Other risk factors include the severity and type of PTEs (Darves-Bornoz et al., 2008), pre-existing mental disorders such as depression and PTSD (Stander et al., 2014) and individual characteristics (Farhood et al., 2016), feeling of guilt (Kubany et al., 1995), and the time interval (Steel et al., 2009) since the PTE. In war-affected populations, torture (Steel et al., 2009), captivity (Farhood et al., 2010), sexual attacks (Wright et al., 2017), experiencing violent attacks (Johansen et al., 2013), and the death of a significant, close person (Tekin et al., 2016) were correlated with PTSD and depression. In a sample of Yazidi women, Kizilhan et al. (2020a,b) found that the number of PTEs experienced and the duration as well as the frequency of captivity predicted comorbid PTSD and depression.

Consequently, the psychosocial stress in these IDP camps is extremely high and many Yazidis suffer from serious mental health problems (Haroz et al., 2018). It was found that 42.9% of Yazidi refugees in Turkish refugee camps suffer from PTSD and 39.5% of major depression (Tekin et al., 2016). In a study with 416 Yazidi women and girls (65 of whom had survived sexual enslavement) in IDP camps in the KRI, more than 80% and almost all survivors who had experienced enslavement fulfilled criteria for PTSD (Ibrahim et al., 2018). It has been shown that formerly enslaved Yazidi females suffer from higher prevalence of mental stress (97.1%), PTSD (90.6%), suicidal ideation (38.1%), depression (36.7%), and general anxiety symptoms (37.4%) (Taha and Slewa-Younan, 2020). Among those who had been sexually abused, very high prevalence rates of mental stress of 50.9% for complex PTSD and 20% for PTSD were found (Hoffman et al., 2018). These high rates, particularly for complex PTSD, emphasise the unique nature of trauma caused by the combination of captivity and sexual slavery. An alternative explanation for the occurrence of complex PTSD might be the stress and poor conditions in the refugee camps after the captivity by ISIS (Hoffman et al., 2018).

Subsequent life events, physical and psychological support, and the role of religion and culture have been shown as important protective factors (Murthy and Lakshminarayana, 2006). Furthermore, daily stressors, such as material deprivation and loss, interparental conflict, abuse, concerns about perceived safety and basic needs, can mediate the relationship between war exposure and mental health (Fernando et al., 2010; Rasmussen et al., 2010).

Suicidal behavior is also an important issue in post-conflict settings (Haroz et al., 2018; Canetto, 2021). Years after a war ended, suicide rates seem to have risen in post-conflict settings (Amowitz et al., 2004; Eytan et al., 2015; Mugisha et al., 2016). For example, surveys conducted in Kosovo 8 years after the war and in Rwanda 14 years after the genocide show that 11% of the Kosovo participants and almost 70% of the Rwanda sample reported that they had already felt suicidal ideation or considered hurting themselves (Eytan et al., 2015). Additionally, Wenzel et al. (2009) reported suicidal ideation to be associated with unemployment, and with high depression and PTSD scores. In post-conflict Northern Uganda, 12.1% stated suicidal ideation and 6.2% suicidal attempts (Mugisha et al., 2016). Suicidality was found to be higher in this Ugandan population when the people suffered from depression and PTSD (Flory and Yehuda, 2015). In Southern Iraq, high attempted-suicide rates of 5 to 7% were found and even higher rates of suicidal ideations of over 20% (Amowitz et al., 2004).

This study investigated the relationship between psychological stress, particularly among Yazidi members living in refugee camps in Iraq and Yazidi members and Non-Yazidi who lived in a more or less secure Kurdistan Region of Iraq during the crimes perpetrated by ISIS.

We excluded those community members with a reported clinical diagnosis of severe mental illness such as a psychosis, which might interfere with informed consent or understanding of the questions in the study instruments. Furthermore, two participants were excluded from the data analysis because they could not be assigned to one of the three groups in terms of their living situation or religion.

## Methods

### Participants, recruiting and sample composition

The sample consists of 425 Yazidi members and Muslim citizens (Sunni Kurds) from the KRI, including 139 Yazidis (67 women and 72 men) from the area of Sinjar, survivors of the 2014 attack by ISIS, living in refugee camps for more than 6 years. 145 Yazidis (78 women and 67 men), originally from the area of Sheikhan, had not been exposed to direct attack by ISIS and thus live near the city of Duhok and were not displaced, threatened or attacked by ISIS. The control group consisted of 141 Sunni Kurds (65 women and 76 men) who live in the area of Dohuk, but not as displaced persons in camps. Being part of a majority in Northern Iraq, they tend to experience comparatively lower levels of discrimination. However, it is important to keep in mind that their social context is complex and many of the Sunni Kurds are nonetheless still exposed to some level of discrimination because of their Kurdish ethnicity, though not of their religious affiliation (Szanto, 2020). The respective relevance of this complex social, religious and ethnic background factors cannot be explored in the limited scope of this paper. The average age of the participants was 29.99 years (*SD* = 10.52) and ranged from 15 to 78 years. Participants were recruited by the University of Dohuk, the government and the management of the refugee camps. The students of IPP do their practical work in the camps for their master studies in psychotherapy and psychotraumatology. For this reason, there has been a close contact with the administration and refugees of the camps since 2015. Informed consent was obtained and all participants took part in the study on a voluntary basis.

### Procedure

The interviews and data collection took place in Dohuk from July 2021 until October 2021, in the place where the participants lived. For the Yazids exposed to violence, this step took place in a room inside the refugee camps, and for the Yazidis not exposed to violence and non-Yazids, it took place in their homes. The interviews were conducted by psychotherapists and psychology master’s students from Dohuk University. They were trained by a research team from the Institute for Transcultural Health Science in Germany *via* zoom. All of the interviewers are fluent in Kurdish and English. Due to the high rate of illiteracy, the interviews were directly and orally translated from English into Kurdish by the interviewers. The Kurdish answers given by the participants were noted down by direct translation into English. Interviews were conducted in a one-to-one setting. Psychologists from the institute for psychotherapy and psychotraumatology at University of Duhok had received specific training in doing the interviews. The study was approved by the ethics commission of the University of Dohuk, no. 032021.

### Study instruments

Demographic data such as gender, age, civil status, number of children and siblings, profession, duration and degree of education, employment, religion, and current living situation were collected by a questionnaire developed for the study together with information on the duration of IS captivity, if experienced. Moreover, information on whether there was a loss of friends or family members, and whether the participants reported being victims of sexual or physical violence, had been collected.

### 36-item short form survey instrument

The 36-item short form survey (SF-36) from Ware Jr and Sherbourne (1992), drawn from the original 245-item Medial Outcomes Study (MOS) questionnaire (Tarlov et al., 1989), was used to survey the general health status. In the validation study of Brazier et al. (1992), the SF-36 demonstrated an internal consistency of *α* ≥ 0.85 and a test–retest reliability with a correlation of *r* ≥ 0.75 for all dimensions except for the dimension social functioning (*α* = 0.73, *r* = 0.74). Good internal consistency and test–retest-reliability were also reported by other studies (Gandek et al., 1998).

### Impact of events scale-revised

To assess psychological trauma and PTSD, the impact of events scale-revised (IES-R) from Weiss and Marmar (1997), the original version of the Impact of Event Scale (Horowitz et al., 1979), which consists of a widely used self-reporting measure within the trauma literature. The IES-R (Weiss and Marmar, 1997) is a 22-item scale and scale scores are formed for the three subscales intrusion, avoidance and hyperarousal to measure the level of symptomatic response to specific traumatic stressors. From these subscales, a possible PTSD can be diagnosed. The internal consistency was shown to be high in all three subscales, this is *α* = 0.87–0.94 for the intrusion scale, *α* = 0.84–0.87 for the avoidance scale and *α* = 0.79–0.91 for the hyperarousal scale (Weiss and Marmar, 1997). The test–retest reliability within 6 months ranged from 0.89 to 0.94 (Weiss and Marmar, 1997).

### Beck depression inventory-II

The beck depression inventory (BDI) was used to measure the presence and degree of depression among the participants (Beck et al., 1961). The most recent version is the beck depression inventory-II (Beck et al., 1996a) which is a widely used upgrade of the amended BDI. It consists of a 21-item multiple choice self-report and can be self-administered or verbally by a trained interviewer (Beck et al., 1996a). The BDI-II demonstrates a high internal consistency of *α* = 0.91 (Beck et al., 1996b) and a high test–retest reliability of *r* = 0.93 (Beck et al., 1996a).

### Beck anxiety inventory

To assess the severity of anxiety among the participants, the Beck Anxiety Inventory (BAI) from Beck et al. (1988) is a self-reporting measure that consists of 21 items. The internal consistency for the BAI is *α* = 0.92 and the test–retest reliability over 1 week is *r* = 0.75 (Beck et al., 1988). Evidence was found for validity criteria (Beck et al., 1988).

### Interpersonal needs questionnaire

The 15-items version of the interpersonal needs questionnaire (INQ-15) from Van Orden et al. (2012) was used to assess suicidality. Originally, the INQ consisted of 25 items (Van Orden et al., 2012). However, the shortened version of 15 items (INQ-15) was chosen as it showed comparatively best psychometric properties (Hill et al., 2015). The subscales (thwarted belongingness and perceived burdensomeness) of the INQ-15 demonstrated high internal consistency (Van Orden et al., 2012). Evidence was found for validity, as both constructs demonstrated convergent associations with related interpersonal constructs (loneliness and social support for the subscale thwarted belongingness and social worth and death ideation for perceived burdensomeness) (Van Orden et al., 2012).

### Statistical analyses

The data set was processed by using IBM SPSS Statistics 27. The significance level was set at *p* < 0.05 for all tests. The data were scored, coded, and analyzed using descriptive statistics and discriminant analysis on the scores of the three groups of according to age, gender, PTSD, depression, suicidality. A critical statistical significance was determined for an overall effect at α = 0.05. Given the size of the survey sample, statistical analyses were carried out with non-parametric statistical methods. To test if there was a difference between the three groups of attacked Yazidis, non-attacked Yazidis and Muslim citizens as regards gender and age, a one factor ANOVA analysis was conducted. Kruskal–Wallis test also includes post-hoc tests (Dunn-Bonferroni tests) to identify differences between the groups. The Spearman’s correlation was used to measure the correlation of the numeric variables age, years of education and number of siblings with perceived burdensomeness. The Eta Coefficient test was used to determine the strength of association between the nominal variables gender, employment, physical violence, loss of important people and flight experience with perceived burdensomeness.

## Results

### Sociodemographic characteristics

The group of the Yazidis exposed to ISIS violence consisted of 139 participants, the group of other Yazidis not exposed to violence of 145 participants and the non-Yazidis of 141 participants (group 3). The numbers and percentages of the sociodemographic characteristics of the three groups and the full sample are represented descriptively in Table 1 for the means and standard deviations for the numerical variables.

**Table 1:** Sociodemographic Characteristics of Participants.

Characteristic	Group 1		Group 2		Group 3		Full sample	
	*n*	*%*	*n*	*%*	*n*	*%*	*n*	*%*
**Gender**								
Female	67	48.2	78	53.8	65	46.1	210	49.4
Male	72	51.8	67	46.2	76	53.9	215	50.6
**Civil status**								
Single	50	36.0	82	56.6	69	48.9	201	47.3
Married	88	63.3	61	42.1	72	51.1	221	52.0
Divorced	1	0.7	2	1.4	0	0.0	3	0.7
Children^a^	81	58.3	53	36.6	60	42.6	194	45.6
Siblings^a^	98	70.5	137	94.5	137	97.2	372	87.5
**Highest educational level**								
Uneducated	68	48.9	23	15.9	13	9.2	104	24.5
Primary School	49	35.3	63	43.4	31	22.0	143	33.6
High School	13	9.4	30	20.7	39	27.7	82	19.3
Apprenticeship	0	0.0	0	0.0	0	0.0	0.0	0.0
Further Education	1	0.7	0	0.0	2	1.4	3	0.7
University	8	5.8	29	20.0	56	39.7	93	21.9
**Employment**								
Unemployed	132	95.0	126	86.9	97	86.8	355	83.5
Employed	7	5.0	19	13.1	44	31.2	70	16.5
ISIS captivity^a^	6	4.3	0	0.0	2	1.4	8	1.9
Victim of sexual violence^a^	5	3.6	1	0.7	1	0.7	7	1.6
Victim of physical violence^a^	44	31.7	30	20.7	2	1.4	76	17.9
Loss of important people^a^	69	49.6	65	44.8	26	18.4	160	37.6
Flight experience^a^	103	74.6	0	0.0	1	0.07	104	24.5

*N* = 425 (*n* = 139 for Group 1, *n* = 145 for Group 2 and *n* = 141 for Group 3). Group 1 = Yazidis who were attacked by ISIS and live in refugee camps now; Group 2 = Yazidis who were not attacked by ISIS and live in a household; Group 3 = Sunni Kurds who live in the same area (control group). ^a^Reflects the number and percentage of participants answering “yes” to this question.

Among the participants, 49.4% were female and 50.5% were male. Of these, 47.3% were single, 52% married, and 0.7% divorced. 45.6% of participants had children and 87.5% had siblings. From the participants 24.5% were uneducated, 33.6% went to primary school, 19.3% to high school, 7% had a further education and 21.9% went to university. Among the participants, 83.5% were unemployed and 1.9% were in IS captivity, 1.6% were victims of sexual violence and 17.9% were victims of physical violence. Within the participants, 37.6% reported having lost at least one significant person in their lives and 24.5% reported having experienced flight. The average age of the participants was 29.99 years old (*SD* = 10.52). The average number of children and siblings which participants had was 1.93 children (*SD* = 2.74) and 5.56 siblings (*SD* = 3.29). Participants had an average mean of 7.78 years of education (*SD* = 5.95), spent an average of 0.04 years in ISIS captivity (*SD* = 0.39) and lost an average of 0.78 important people in their life (*SD* = 1.77) (Table 2).

**Table 2:** Means and standard deviations of sociodemographic characteristics of participants for numerical variables.

Characteristic	Group 1		Group 2		Group 3		Full sample	
	*M*	*SD*	*M*	*SD*	*M*	*SD*	*M*	*SD*
Age	31.92	12.06	27.74	9.93	30.40	9.01	29.99	10.52
Number of children	2.88	3.44	1.52	2.33	1.43	2.03	1.93	2.74
Number of siblings	3.78	3.19	6.60	3.27	6.25	2.65	5.56	3.29
Years of education	3.79	4.41	8.19	5.33	11.30	5.51	7.78	5.95
ISIS captivity (years)	0.12	0.65	0.00	0.00	0.02	0.19	0.04	0.39
Loss of important people (number)	1.22	2.60	0.86	1.34	0.27	0.74	0.78	1.77

*N* = 425 (*n* = 139 for Group 1, *n* = 145 for Group 2 and *n* = 141 for Group 3). Group 1 = Yazidis who were attacked by ISIS and live in refugee camps now; Group 2 = Yazidis who were not attacked by ISIS and live in a household; Group 3 = Sunni Kurds who live in the same area (control group). M, mean; SD, standard deviation.

Yazidis who exposed to violence shows the highest percentage for unemployment, exposure to sexual and physical violence, ISIS captivity (though only 1, 9% as most were exposed outside of captivity), had suffered the loss of important persons most frequently and had the lowest education level in comparison with the other Yazidis not exposed to violence and non-Yazidis.

### Physical and mental health between the three groups

The descriptive statistics for the psychological stress of the three groups can be seen in Table 3. The results of the differences of general physical health, general mental health, level of depression, level of anxiety, intrusion, avoidance, hyperarousal, perceived burdensomeness and thwarted belongingness between the three groups are presented in Table 4.

**Table 3:** Means and standard deviations of psychological stress and suicidality of the three groups.

Measure	Group 1		Group 2		Group 3	
	*M*	*SD*	*M*	*SD*	*M*	*SD*
Depression	14.20	0.990	10.26	0.776	13.26	0.893
Anxiety	7.22	0.498	3.15	0.346	6.94	0.491
**PTSD**						
Intrusion	11.67	0.738	3.30	0.606	3.32	0.611
Avoidance	9.71	0.666	3.59	0.660	3.26	0.619
Hyperarousal	6.82	0.494	2.32	0.443	2.29	0.422
Physical health	46.39	0.981	49.72	0.980	51.09	0.738
Mental health	38.50	0.695	45.54	0.916	35.84	0.989
**Suicidality**						
Perceived burdensomeness	1.65	0.098	1.45	0.113	1.49	0.080
Thwarted belongingness	3.35	0.077	2.65	0.112	3.75	0.077

*N* = 425 (*n* = 139 for Group 1, *n* = 145 for Group 2 and *n* = 141 for Group 3). Group 1 = Yazidis who were attacked by ISIS and live in refugee camps now; Group 2 = Yazidis who were not attacked by ISIS and live in a household; Group 3 = Sunni Kurds who live in the same area (control group). M, mean; SD, standard deviation.

**Table 4:** Values and significance of the non-parametric Kruskal–Wallis test and Dunn–Bonferroni *post-hoc* tests for the comparisons between the three groups for all measures.

Measure	Kruskal–Wallis test (*χ^2^*)	Group 1 versus Group 2	Group 1 versus Group 3	Group 2 versus Group 3
Depression	7.983*	2.591*	0.341	−2.255
Anxiety	52.167***	6.398***	0.356	−6.062***
**PTSD**				
Intrusion	108.124***	9.354***	8.658***	−0.638
Avoidance	98.032***	8.725***	8.458***	−0.209
Hyperarousal	100.724***	9.049***	8.330***	−0.663
Physical health	10.487**	−2.555*	−3.010**	−0.478
Mental health	53.912***	−5.247***	1.767	7.051***
**Suicidality**				
Perceived burdensomeness	22.725***	4.721***	1.827	−2.893*
Thwarted belongingness	69.252***	4.340***	−3.920***	−8.317***

*N* = 425 (*n* = 139 for Group 1, *n* = 145 for Group 2 and *n* = 141 for Group 3). Group 1, Yazidis who were attacked by ISIS and live in refugee camps now; Group 2, Yazidis who were not attacked by ISIS and live in a household; Group 3, Sunni Kurds who live in the same area (control group). *p < 0.05. **p < 0.01. ***p < 0.001.

### General physical health

The Kruskal–Wallis test showed that there was a statistically significant difference in the total score of general physical health between the three groups, *χ^2^*(2) = 10.487, *p* = 0.005, with a mean rank score of 185.63 for group 1, 222.88 for group 2, and 229.82 for group 3. A higher score represents a more favorable health state. Subsequent post-hoc tests (Dunn-Bonferroni tests) show that only groups 1 and 2 (*z* = −2.555, *p* = 0.032), and 1 and 3 (*z* = −3.010, *p* = 0.008) differ significantly, but not groups 2 and 3. The results indicate that the score for general physical health was significantly lower by Yazidis exposed to violence compared to Yazidis not exposed to violence and non-Yazidis. The effect between Yazidis exposed violence and Yazidis not exposed violence is weak with *r* = 0.15.

### Mental health

The Kruskal–Wallis test showed that there was a statistically significant difference in the total score of general mental health between the three groups, *χ^2^*(2) = 53.912, *p* < 0.001, with a mean rank score of 195.50 for Yazidis exposed violence, 272.01 for Yazidis not exposed violence, and 169.57 for non-Yazidis. A higher score represents a more favorable health state. Subsequent post-hoc tests (Dunn-Bonferroni tests) show that only by Yazidis exposed violence and Yazidis not exposed violence (*z* = −5.247, *p* < 0.001), and Yazidis not exposed violence and non-Yazidis (*z* = 7.051, *p* < 0.001) differ significantly, but not Yazidis exposed violence and on-Yazidis. It can be assumed that the score for general mental health was significantly lower in Yazidis exposed violence and non-Yazidis compared to Yazidis not exposed violence. The effect between Yazidis exposed violence and Yazidis not exposed violence is medium with *r* = 0.31.

### Depression

The Kruskal–Wallis test showed that there was a statistically significant difference in the total score of the BDI-II to measure the level of depression between the three groups, *χ^2^*(2) = 7.983, *p* = 0.018, with a mean rank score of 227.53 for Yazidis exposed violence, 189.81 for Yazidis not exposed violence, and 222.52 for non-Yazidis. Subsequent post-hoc tests (Dunn-Bonferroni tests) show that only Yazidis exposed violence and Yazidis not exposed violence (*z* = 2.591, *p* = 0.029) differ significantly, but not Yazidis exposed violence and non-Yazidis, nor Yazidis not exposed violence and non-Yazidis. It can be assumed that the BDI-II score for the level of depression was significantly higher in Yazidis exposed violence than by Yazidis not exposed violence, but not significantly higher compared to non-Yazdis. The effect between group Yazidis exposed violence and Yazidis not exposed violence is weak with *r* = 0.15.

### Anxiety

The Kruskal–Wallis test showed that there was a statistically significant difference in the score of the selected items of the BAI to measure the level of anxiety between the three groups, *χ^2^*(2) = 52.167, *p* < 0.001, with a mean rank score of 246.18 for Yazidis exposed violence, 153.95 for Yazidis not exposed violence, and 241.01 for non-Yazidis. Subsequent post-hoc tests (Dunn-Bonferroni tests) show that only Yazidis exposed violence and Yazidis not exposed violence (*z* = 6.398, *p* < 0.001), and Yazidis not exposed violence and non-Yazidis (*z* = −6.062, *p* < 0.001) differ significantly, but not Yazidis exposed violence and non-Yazidis. It can be assumed that the score for the level of anxiety was significantly lower by Yazidis not exposed violence and non-Yazids compared to Yazidis not exposed violence. The effect between Yazidis exposed violence and Yazidis not exposed violence is medium with *r* = 0.38.

### Trauma: Intrusion, avoidance and hyperarousal

Based on ICD-11, we collected Instrusion, Avoidence, and Hypererausal from the samples. For prevelence of PTSD in Yazidis after the 2014 genocide, we refer to Tekin et al. (2016), Cetorelli et al. (2017), Ibrahim et al. (2018), and Kizilhan et al. (2020a,b).

The Kruskal–Wallis test showed that there was a statistically significant difference in the total score of the subscale intrusion between the three groups, *χ^2^*(2) = 108.124, *p* < 0.001, with a mean rank score of 292.15 for Yazidis exposed violence 1, 170.45 for Yazidis not exposed violence, and 178.72 for non-Yazidis. Subsequent post-hoc tests (Dunn-Bonferroni tests) show that only Yazidis exposed violence and Yazidis not exposed violence (*z* = 9.354, *p* < 0.001), and Yazidis exposed violence and non-Yazidis (*z* = 8.658, *p* < 0.001) differ significantly, but not Yazidis not exposed violence and non-Yazidis. The subscale avoidance between the three groups, *χ^2^*(2) = 98.032, *p* < 0.001 was significant with a mean rank score of 288.14 for Yazidis exposed violence, 175.15 for Yazidis not exposed violence, and 177.84 for non-Yazidis. Subsequent post-hoc tests (Dunn-Bonferroni tests) show that only Yazidis exposed violence and Yazidis not exposed violence (*z* = 8.725, *p* < 0.001), and Yazidis exposed violence and non-Yazidis (*z* = 8.458, *p* < 0.001) differ significantly, but not groups Yazidis not exposed violence and non-Yazidis. It can be assumed that the total score for the subscale avoidance is significantly higher by Yazidis exposed violence compared to Yazidis not exposed violence and non-Yazidis.

The subscale hyperarousal between the three groups, *χ^2^*(2) = 100.724, *p* < 0.001, with a mean rank score of 289.25 for Yazidis exposed violence, 171.71 for Yazidis not exposed violence, and 180.29 for non-Yazidis. Subsequent post-hoc tests (Dunn-Bonferroni tests) show that only by Yazidis exposed violence and Yazidis not exposed violence (*z* = 9.049, *p* < 0.001), and Yazidis exposed violence and non-Yazidis (*z* = 8.330, *p* < 0.001) differ significantly, but not groups Yazidis not exposed violence and non-Yazidis. It can be assumed that the total score for the subscale hyperarousal is significantly higher by Yazidis not exposed violence compared to Yazidis not exposed violence and non-Yazidis. The effect between Yazidis exposed violence and Yazidis not exposed violence is strong with *r* = 0.54/52/56.

### Perceived and thwarted burdensomeness

The Kruskal–Wallis test showed that there was a statistically significant difference in the total score of perceived burdensomeness between the three groups, *χ^2^*(2) = 22.725, *p* < 0.001, with a mean rank score of 239.68 for Yazidis exposed violence, 182.97 for Yazidis not exposed violence, and 217.59 for non-Yazidis. Subsequent post-hoc tests (Dunn-Bonferroni tests) show that only by Yazidis exposed violence and Yazidis not exposed violence (*z* = 4.721, *p* < 0.001), and Yazidis not exposed violence and non-Yazidis (*z* = −2.893, *p* = 0.011) differ significantly, but not Yazidis exposed violence and non-Yazidis. It can be assumed that the score for perceived burdensomeness to measure suicidality was significantly higher by Yazidis exposed violence and non-Yazidis compared to Yazidis not exposed violence. The effect between Yazidis exposed violence and Yazidis not exposed violence is almost medium with *r* = 0.28 whereas the effect between Yazidis not exposed violence and non-Yazidis is weak with *r* = 0.17.

The Kruskal–Wallis test showed that there was a statistically significant difference in the total score of thwarted belongingness between the three groups, *χ^2^*(2) = 69.252, *p* < 0.001, with a mean rank score of 215.49 for Yazidis exposed violence, 152.29 for Yazidis not exposed violence, and 272.98 for non-Yazidis. Subsequent post-hoc tests (Dunn-Bonferroni tests) show that all groups differed significantly, Yazidis exposed violence and Yazidis not exposed violence (*z* = 4.340, *p* < 0.001), Yazidis not exposed violence and non-Yazidis (*z* = −8.317, *p* < 0.001), and Yazidis exposed violence and non-Yazidis (*z* = −3.920, *p* < 0.001). It can be assumed that the score for thwarted belongingness to measure suicidality was significantly higher by Yazidis exposed violence compared to Yazidis not exposed violence, and even higher in non-Yazidis compared to Yazidis exposed violence. The effect between Yazidis exposed violence and Yazidis not exposed violence is almost medium with *r* = 0.26, the effect between Yazidis exposed violence and non-Yazidis is weak with *r* = 0.23, and the effect between Yazidis not exposed violence and non-Yazidis is almost strong with *r* = 0.49.

### Socioeconomic effects on suicidality

Spearman’s correlation, eta coefficient tests and multiple regression analyses were conducted to measure if the socioeconomic variables age, years of education, gender, employment, physical violence, loss of important people, flight experience and number of siblings correlate with suicidality, namely perceived burdensomeness and thwarted belongingness.

As the assumptions of a multiple regression analysis for the variable perceived burdensomeness were not met (Eid et al., 2017), a non-parametric Spearman’s correlation was run to determine the relationship between participants’ age and perceived burdensomeness, the duration of education in years and perceived burdensomeness, and the number of siblings and perceived burdensomeness. There was a small negative correlation between the duration of education and perceived burdensomeness, which was statistically significant with *r_s_* (423) = −0.102, *p* = 0.036. The fewer years of education a participant had, the higher was the score for perceived burdensomeness. There was no significant correlation between participants’ age and perceived burdensomeness, nor between number of siblings and perceived burdensomeness as can be seen in Table 5.

**Table 5:** Descriptive statistics and Spearman’s correlation for perceived burdensomeness, age, education in years, and number of siblings.

Variable	*Mdn*	*SD*	1	2	3	4
1. Perceived burdensomeness	1.00	1.17	-			
2. Age	28.00	10.52	0.007	-		
3. Education in years	8.00	5.95	−0.102*	−0.229**	-	
4. Number of siblings	6.00	3.29	−0.029	0.076	0.099*	-

*p < 0.05. **p < 0.01. Mdn, median; SD, standard deviation.

## Discussion

The aim of this study was to investigate the relationship between psychological stress among members of the Yazidi community 7 years after the genocide perpetrated by ISIS in Iraq in 2014. Three groups were analyzed with regard to their physical and mental health: (1) Yazidis who experienced violence at the hands of ISIS and now live in refugee camps in the KRI, (2) Yazidis who were not attacked by ISIS and do not live in refugee camps and (3) a control group of Muslim citizens who live in the same area but tend to experience comparatively lower levels of discrimination. Psychological stress and suicidality were measured and compared in the three different groups. Furthermore, the relationship between socioeconomic factors and suicidality was examined.

Significant differences in psychological stress (general physical health, general mental health, level of depression, level of anxiety, subscales of PTSD such as intrusion, avoidance and hyperarousal) were found between the three groups. Post-hoc comparisons (Dunn-Bonferroni tests) for the Kruskal–Wallis test show that the Yazidis exposed violence by ISIS reached higher scores for perceived burdensomeness and thwarted belongingness than Yazidis who were not attacked by ISIS. Interestingly, the scores for perceived burdensomeness of attacked Yazidis and Muslim citizens did not differ significantly, and Muslim citizens reached even higher scores for thwarted belongingness than the group of the attacked Yazidis.

The Yazidis exposed violence also showed lower scores for general physical health, general mental health, and higher scores for level of depression, level of anxiety, subscales of PTSD such as intrusion, avoidance and hyperarousal in comparison with the Yazidis not exposed violence. However, Yazidis exposed violence by ISIS did not always have a significantly higher (or lower) score than the non-Yazidis. The Yazidis not exposed violence reached lower scores for general physical health, higher scores for intrusion, avoidance and hyperarousal. However, this was not the case for general mental health, level of depression and level of anxiety, as the Yazidis exposed violence always had higher psychological stress scores (or lower scores for general physical and mental health) than the Yazidis not exposed violence, but not always higher scores (or lower scores for general physical and mental health) than non-Yazidis.

Further analyses were conducted to measure the relationship between socioeconomic variables – such as age, years of education, gender, employment, physical violence, loss of important people, flight experience, number of siblings - and suicidality. There was a small negative correlation between the duration of education and perceived burdensomeness, which was statistically significant. The fewer years of education a participant had, the higher was the score for perceived burdensomeness. Moreover, it was found that employment significantly predicts thwarted belongingness, as does physical violence and loss of important people.

As the results show, the Yazidis who were attacked by ISIS always had higher scores for suicidality and psychological stress compared to the Yazidis not exposed violence, but not always compared to the non-Yazidis. The score differences were especially high for PTSD. These results are to be expected and understandable to a large extent, as Yazidis exposed violence has experienced by far most stressors compared to Yazidis not exposed violence and non-Yazidis. Many of them have experienced sexual or physical violence, had to flee, been in ISIS captivity, lost important people in their lives, generally have low levels of education, and are mostly unemployed. All the mentioned factors could explain why these individuals are at higher risk for psychological stress and suicidality. Furthermore, the higher risk might be increased additionally by difficult living conditions in refugee camps (Yazda, 2017), the current COVID-19 pandemic (Kizilhan and Noll-Hussong, 2020) and other daily stressors (Fernando et al., 2010; Rasmussen et al., 2010).

However, it is interesting to observe that non-Yazidis scored the highest on thwarted belongingness and the same as Yazidis exposed violence on perceived burdensomeness. Participants of non-Yazidis are Kurdish Sunni Muslims and, themselves, represent an ethnic minority in Iraq (Mohamed, 2014). They make up only 16% of the Iraqi population, whereas the majority of 78% consists of Arabs. Whilst the majority of Iraqi Kurds are Sunni Muslims, 62% of Iraqi Arabs are Shia Muslims and 30% Sunni Muslims (Mohamed, 2014). Although they are not a minority in Iraqi Kurdistan, they are still a religious and ethnical minority in relation to the whole population in the country. There has been a lot of tension and fighting between Kurds, Arab Shias and Arab Sunnis (Szanto, 2020). Especially, the hostility between Kurds and Arab Shias has increased over the last years due to the opposition of the Shia government to the referendum on Kurdish independence and the Kurds blaming the Shia for the alienation of Sunni Arabs and the rise of ISIS. Furthermore, the genocide against the Kurds forced many of them to flee and a lot took on new groups of religion which are sometimes anti-Shia (Szanto, 2020). This has further increased the prior split between the two groups. These factors could explain the high scores of the non-Yazidis for thwarted belongingness. It is reasonable that after experiencing such persecution and hostility, non-Yazidis is still marginalized and does not feel that they belong properly to the society they are living in, nor do they feel valued and appreciated within the country. Moreover, all these conflicts could also have an influence on the perceived burdensomeness which would explain why non-Yazidis did not show significantly lower scores on this variable.

All participants, both Yazidi and Muslims, scored low on perceived burdensomeness. One explanation could be that people from this culture generally think in a more collective way in which personal feelings and emotions are not considered as so important (Kizilhan, 2017b).

Moreover, there is a different understanding of mental illness, whereby it is seen as a consequence of fate and not really as an interaction of various factors (Kizilhan, 2017a). This fact can lead to a stigmatization of mental illness problems. All these aspects can explain why the participants might not have been able to adequately express their feelings and emotions about their perceived burdensomeness, as the collective society is regarded to be more important than the individual well-being. Moreover, suicidality might also be a taboo subject in this society (e.g., Jäger et al., 2019), thereby further constraining individuals and thus perpetuating the vicious circle.

Physical violence is often associated with trauma and PTSD (Resnick et al., 1993; Kessler et al., 1995), which can lead to a disordered worldview. Similarly, the fact of losing important people in life may damage the sense of belongingness as well as the experience of being valued. This is due to the fact that they substantially contribute to the feeling of fitting into the broader social context.

We found a direct relationship between physical violence, loss of important people, flight experience, duration of education, gender and perceived burdensomeness. These are all factors that increase the perceived burdensomeness at different levels. Being a woman in the KRI is associated with many obstacles, less freedoms and more restrictions (Joly and Bakawan, 2016). There is a deeply-established gender inequality between men and women in Iraq which is also supported by the Iraqi law (Joly and Bakawan, 2016). Patriarchal norms and traditions further increase men’s domination over women (Joly and Bakawan, 2016) and the prevalence of intimate partner violence is very high and has significant consequences in terms of serious injuries (Al-Atrushi et al., 2013). Women are seen as property of the family and often represent the symbol of a family’s honor and dignity in terms of purity, virginity and modesty (Laizer, 1991). These circumstances could lead to a culture in which women perceive themselves increasingly as a burden to others by feeling that they are not important enough and therefore represent a burden to society. This, in turn, would constitute an additional challenge for their family or society.

There are countries in which women have higher suicide rates than men and therefore represent an exception to this gender paradox. Examples include China, Pakistan, Bangladesh, Indonesia, Nepal, and also Iraq. It has been shown in several previous findings that there is an association between violence and suicidality (Devries et al., 2011), intimate, physical and emotional partner violence and suicidal behavior (Weaver et al., 2007), and also between rape and suicidality. In Kurdistan, women are often victims of intimate partner violence (Al-Atrushi et al., 2013). For this reasons, physical violence could be a mediator between gender and suicidality in the context of women in the KRI. It may lead to new insights to carry out further mediating analyses to examine the link between gender, physical violence and suicidality.

### Limitations and future research

The present study has some limitations. In connection to the survey, it can be criticized that all variables were recorded by means of self-reporting interviews and, consequently, only the subjective point of view of the participants was recorded. To address this issue, future studies could additionally include objective survey methods such as a third-party evaluation to guarantee more objective and valid results. However, it has to be considered that access to psychological treatment in refugee camps is very limited. For this reason, it is difficult to obtain objective third-party reports in the context at hand. Furthermore, a limitation is that the interviews had to be translated for the participants, of which many are illiterate. Interviews were conducted and translated by psychotherapists and psychology master’s students from Dohuk University. The interviews are not validated in Kurdish, so they were translated orally from English to Kurdish. This can lead to inaccurate translations and comprehension problems as well as different interpretations of the interviewers. In connection to that, the different culture and understanding of illness or disorder concepts must also be considered.

For future research, it would be interesting to add a follow-up study to measure the development of suicidality and psychological stress scores across time. In that context, the influence of the COVID-19 pandemic could be measured to see to what extent it affects the mental health state of people in refugee camps in the KRI.

Considering the culture and religion including the caste system of the Yazidis, important and relevant topics such as shame and historical trauma (Kizilhan et al., 2020b) and fear of exclusion (Kizilhan and Noll-Hussong, 2017) could be examined and put into relation with mental health.

## Data availability statement

The datasets presented in this study can be found in online repositories. The names of the repository/repositories and accession number(s) can be found in the article/supplementary material.

## Ethics statement

The studies involving human participants were reviewed and approved by University of Duhok IPP. The patients/participants provided their written informed consent to participate in this study.

## Author contributions

JK planned the study and conducted the study with support of his research team. LS analyzed the data and wrote the draft manuscript with support from JK. LS was supervised by TB. TW contributed by revising the manuscript and reanalyze some of the data. All authors contributed to the article and approved the submitted version.

## Conflict of interest

The authors declare that the research was conducted in the absence of any commercial or financial relationships that could be construed as a potential conflict of interest.

## Publisher’s note

All claims expressed in this article are solely those of the authors and do not necessarily represent those of their affiliated organizations, or those of the publisher, the editors and the reviewers. Any product that may be evaluated in this article, or claim that may be made by its manufacturer, is not guaranteed or endorsed by the publisher.
